# Effects of cyclic adenosine monophosphate modulators on maturation and quality of vitrified-warmed germinal vesicle stage mouse oocytes

**DOI:** 10.1186/s12958-020-0566-8

**Published:** 2020-01-20

**Authors:** Dayong Lee, Hyang Heun Lee, Jung Ryeol Lee, Chang Suk Suh, Seok Hyun Kim, S. Samuel Kim

**Affiliations:** 1 Department of Obstetrics and Gynecology, Seoul National University Bundang Hospital, 82 Gumi-ro 173 Beon-gil, Seongnam, Gyeonggi-do 13620 South Korea; 2 Department of Obstetrics and Gynecology, Seoul National University College of Medicine, Seoul, South Korea; 3 Department of Obstetrics and Gynecology, University of Kansas Medical Center, Kansas City, KS USA; 4 Eden Centers for Advanced Fertility, Fullerton, CA USA

**Keywords:** Cyclic adenosine monophosphate, Modulator, Vitrification, Germinal vesicle, Oocyte

## Abstract

**Background:**

It is still one of the unresolved issues if germinal vesicle stage (GV) oocytes can be successfully cryopreserved for fertility preservation and matured in vitro without damage after warming. Several studies have reported that the addition of cyclic adenosine monophosphate (cAMP) modulators to in vitro maturation (IVM) media improved the developmental potency of mature oocytes though vitrification itself provokes cAMP depletion. We evaluated whether the addition of cAMP modulators after GV oocytes retrieval before vitrification enhances maturation and developmental capability after warming of GV oocytes.

**Methods:**

Retrieved GV oocytes of mice were divided into cumulus-oocyte complexes (COCs) and denuded oocytes (DOs). Then, GV oocytes were cultured with or without dibutyryl-cAMP (dbcAMP, cAMP analog) and 3-isobutyl-l-methylxanthine (phosphodiesterase inhibitor) during the pre-vitrification period for 30 min.

**Results:**

One hour after warming, the ratio of oocytes that stayed in the intact GV stage was significantly higher in groups treated with cAMP modulators. After 18 h of IVM, the percentage of maturation was significantly higher in the COC group treated with dbcAMP. The expression of F-actin, which is involved in meiotic spindle migration and chromosomal translocation, is likewise increased in this group. However, there was no difference in chromosome and spindle organization integrity or developmental competence between the MII oocytes of all groups.

**Conclusions:**

Increasing the intracellular cAMP level before vitrification of the GV oocytes maintained the cell cycle arrest, and this process may facilitate oocyte maturation after IVM by preventing cryodamage and synchronizing maturation between nuclear and cytoplasmic components. The role of cumulus cells seems to be essential for this mechanism.

## Introduction

Oocyte cryopreservation is an important method in infertility treatment as well as fertility preservation [1]. Since the vitrification method has been introduced and related technologies have been developed, the survival or developmental competence of cryopreserved oocytes is comparable to non-vitrified oocytes [2]. However, ovarian stimulation is required to obtain a sufficient number of mature oocytes. In this procedure, various medications including gonadotropins should be administered and the growth of follicles must be monitored, which is costly, time-consuming, and accompanied by the risk of side effects such as ovarian hyper-stimulation syndrome. These disadvantages can be more pronounced in cases such as young cancer patients, who are unable to delay treatment.

One of the ways to overcome these drawbacks is to retrieve oocytes of germinal vesicle (GV) stage. GV oocyte retrieval has some advantages including; 1) no or minimal administration of exogenous gonadotropin and associated drugs to stimulate oocyte growth and ovulation, 2) fewer side effects associated with ovarian hyper-stimulation, 3) less monitoring of follicle growth, 4) completing treatment within few days, 5) retrieving oocytes regardless of the menstrual cycle, even in the luteal phase, and 6) avoiding the use of hormones on hormone-sensitive cancer patients [3]. With these advantageous, GV oocyte retrieval could be a more favorable modality in terms of cost and patients’ comfort.

After GV oocytes are retrieved, they can be developed into mature oocyte through in vitro maturation (IVM), and acquired MII oocytes can be cryopreserved. It is unclear whether all the frozen mature oocytes will be used for fertilization. This is because if infertility treatment is successful and pregnancy occurs, the remaining frozen mature oocytes may not be needed anymore. In another strategy, GV oocytes can be cryopreserved immediately after retrieval and some of them can be warmed at the time of infertility treatment. This approach has the advantage of reducing the time, effort, and cost which are required for IVM of all GV oocytes. The survival of the cryopreserved GV oocytes after warming has improved to comparable levels as the MII stage oocytes owing to the development of vitrification techniques. However, the maturation rate of warmed GV oocytes and the developmental competence after fertilization are significantly lower than those of vitrified MII oocytes acquired through IVM. Therefore, it is still recommended to freeze MII oocytes rather than GV oocytes [4].

In order to overcome this disadvantage, studies to improve the maturation and developmental competence of GV oocytes that have been warmed after vitrification were carried out. Several studies on humans and animal models have shown that oocyte growth and development are enhanced by the regulation of meiotic resumption. There are some studies on humans or animals to delay or prevent spontaneous meiotic resumption in IVM process with C-type natriuretic peptide (CNP) [5, 6] or chemicals including cyclic adenosine monophosphate (cAMP) analog, kinase or phosphodiesterase inhibitors [7–11]. Yang et al. supplemented CNP to the culture medium during IVM of mice vitrified-warmed cumulus-oocyte complexes (COCs), and the developmental competence of oocyte was ameliorated [6]. Ezoe et al. reported that the addition of cAMP modulators to IVM media improved the developmental competence of vitrified-warmed GV oocytes in bovine [11]. In that study, the cAMP level in the oocyte was drastically reduced after the vitrification process, though no existing studies have considered this aspect.

In mammalian oocyte meiosis, cytoskeleton dynamics plays a crucial role. The meiotic spindle is the major structure involved with the segregation of chromosomes, and dislocation of the spindle increases the risk of errors in chromatid segregation which result in fertilization failure and other developmental anomalies [12]. Meiotic spindle migration and chromosomal translocation occurs through the cytoskeleton consist of F-actin and microtubules [13].

It is unknown if the increasing of the cAMP level with cAMP modulators just after GV oocyte retrieval can maintain the meiotic arrest of GV oocyte and improve maturation after vitrification. The objective of the present study is to assess the effects of cAMP modulators on meiotic arrest of GV oocytes and maturation of GV oocytes after vitrification and warming. In this process we evaluated the cytoskeleton involved in spindle migration and chromosomal translocation.

## Materials and methods

We used two types of cAMP-modulating agents: dibutyryl-cAMP (dbcAMP, Sigma, St. Louis, MO, USA) and 3-isobutyl-l-methylxanthine (IBMX, Sigma). Several studies on humans and animal models have shown that oocyte growth and development are enhanced by regulation of meiotic resumption through raising cAMP levels in oocytes using cAMP modulators [14–17]. Among cAMP modulators, dbcAMP (cAMP analog), and IBMX (non-specific phosphodiesterase inhibitor) were applied as the most frequently adopted representative modulators.

### Immature oocytes collection

Immature oocytes were obtained from 6-week-old female BD-F1 mice (Orient Co., Seoul, South Korea). All experiments were performed in accordance with the institutional guidelines established by the Animal Care and Use Committee of Seoul National University Bundang Hospital. Immature oocytes from ovaries can be retrieved after gonadotropin priming or without ovarian stimulation. Previous studies on humans have suggested that immature oocytes retrieved from stimulated oocytes are more affected by vitrification [18–20] than from immature oocytes obtained from unstimulated oocytes [21, 22]. Reflecting these results, immature oocytes were collected after oocyte stimulation. Immature oocytes were obtained from mice previously treated with intraperitoneal injection with 7.5 IU pregnant mare’s serum gonadotropin (PMSG, Sigma). The mice were killed by cervical dislocation, the mouse ovaries were collected in collection medium (Leibovitz’s, L-15, Gibco, Grand Island, NY, USA) supplemented with 5% heat-inactivated fetal bovine serum (FBS, Invitrogen, Carlsbad, CA, USA).

GV oocytes are supplied with cAMP through gap-junctions from surrounding cumulus cells. We planned to examine the effects of intact gap-junction as an additional cAMP supply from surrounding cumulus cells through the effect of cAMP-modulating agents. For this purpose, only cumulus-oocyte complexes (COCs) with intact cumulus layers at the time of retrieval were selected, and denuded oocytes (DOs) at retrieval were discarded. All oocytes in DO group in this experiment are mechanically denuded from COCs. Mechanical denudation of cumulus cells was processed to disconnect the gap-junction. To proceed with the denudation as soon as collecting COCs, each mouse was randomly assigned into COC group and mechanically DO group before oocyte retrieval. In both groups, follicles were mechanically isolated with a 25-G needle, and COCs were retrieved by puncturing the antral follicles. In the DO group, denudation was proceeded by repeated pipetting and flushing through a controlled fine bore pipette.

### cAMP modulator treatment before IVM

The retrieved immature oocytes from each mouse were washed three times in tissue culture media (M-199, Gibco) containing 10% FBS. Then, these GV oocytes were cultured with or without dbcAMP and IBMX during the pre-vitrification period for 30 min. In accordance with previous mouse studies, the concentrations of dbcAMP and IBMX used were 100 μg/ml and 200 μg/ml, respectively [23–25].

### Vitrification of oocytes at germinal vesicle stage and warming

The immature oocytes were suspended in an equilibrium solution (7.5% ethylene glycol (EG), 7.5% 1,2-propanediol (PROH), and 20% FBS in HEPES-buffered TCM-199 medium) for 5 min. The oocytes were then re-suspended in vitrification solution (15% EG, 15% PROH, 0.5 M sucrose, and 20% FBS in TCM-199) for 45–60 s at room temperature. Two oocytes were loaded onto a CryoTop (Kitazato, Tokyo, Japan), which was then immediately plunged into liquid nitrogen for long term storage. For warming, the CryoTop was immersed directly in a 37 °C warming solution (1.0 M sucrose in 20% FBS in HEPES-buffered TCM-199 medium) for 1 min. The warmed oocytes were transferred to 0.5, 0.25, and then 0 M sucrose in HEPES-buffered TCM-199 medium containing 20% FBS for 3 min each. Oocytes were transferred to the culture medium at 37 °C in humidified air with 5% CO2.

### In vitro maturation

After warming, GV oocytes were matured in maturation medium for 18 h. Maturation medium was composed of 75 mIU/ml recombinant FSH, 0.5 IU/ml hCG (Serono, Geneva, Switzerland), 1% ITS (Sigma), 10 ng/ml recombinant epidermal growth factor (Sigma), and 10% FBS in TCM-199 medium.

### Immunohistochemistry

GV oocytes were fixed 1 h after warming and chromatin integrity was assessed. All other immunohistochemistry was performed on MII oocytes 18 h after IVM. The effects of cAMP modulators on microstructures involved in meiosis were assessed in groups of six including controls. The α-tubulin which constitutes the spindle fiber was stained to compare the chromosome and spindle integrity of the MII oocytes. Bipolar spindle with chromosomes aligned along the equatorial plate was considered normal according to previous studies [12]. The expression of F-actin was measured which is involved in meiotic spindle migration and chromosomal translocation.

The immunostaining procedure was performed as described in a previous study [26]. The oocytes were fixed in 4.0% paraformaldehyde in phosphate-buffered saline (PBS) for 30 min at 4 °C. To permeabilize and block the fixed oocytes, they were incubated in a solution with 0.1% Triton X-100, 0.05% Tween-20 and 5% BSA. After washing, oocytes were incubated overnight in each primary antibody (α-tubulin; diluted to 1:300, F-actin; diluted to 1:1000) in PBS with 0.5% BSA. Following an additional wash, oocytes were incubated with a second antibody (anti-rabbit goat secondary antibody, Molecular Probes, Eugene, OR, USA) conjugated with Alexa Flour-488 or − 594 (diluted to 1:100). After washing, oocytes were mounted using Vectashield (Vector Laboratories, Burlingame, CA, USA) containing 0.5 μg 4,6-diamidino-2-phenylindole (DAPI). Localization of each antibody revealed by FITC and DAPI fluorescence was observed under 400× magnification with Carl Zeiss fluorescence laser confocal microscope ZEN2011 software (LSM710, Carl Zeiss, Germany). Quantification of average fluorescence levels between groups was conducted using ImageJ pixel intensity analysis, and 15–27 oocytes in each group were analyzed.

The oocytes at the early stage of IVM were classified into three groups: intact GV, pre-MI, and MI oocytes. An MII oocyte was classified as normal if it had a barrel-shaped bipolar spindle with distinct, tightly aligned chromosomes on the metaphase plate and well-organized microtubule fibers. Oocytes with an abnormal chromatin configuration were classified as degenerated.

### Statistical analysis

The statistical software package SPSS version 22.0 (SPSS Inc., Chicago, IL) and GraphPad Prism 6.0 (GraphPad Software, La Jolla, CA) was used for analysis. Data are presented as means for continuous variables and percentages for categorical variables. We performed the one way ANOVA to compare continuous variables between 6 groups. When ANOVA indicated a significant difference (*p* < 0.05), Tukey’s HSD post hoc was used. The Chi-squared test was performed to compare proportions. The results were considered statistically significant if the *p*-value was less than 0.05.

## Results

### Survival and maturation after IVM

After 18 h of IVM, there was no difference in the survival rate between the COC groups and the DO groups, and no difference was observed in the groups treated with the cAMP modulator. In contrast, the percentage of maturation after 18 h of maturation was significantly higher in the COC groups than in the DO groups (Table 1). Among the COC groups, the proportion of oocytes that developed to MII oocytes was significantly higher in the GV oocytes group treated with dbcAMP than in the other groups. However, in the DO groups, the percentage of maturation was significantly reduced in the GV oocytes group treated with dbcAMP. In the IBMX-treated groups, no significant difference was observed compared to the control groups. Thus, we found that the addition of dbcAMP in the presence of cumulus cells improves the maturation of vitrified-warmed GV oocytes after IVM.

**Table 1:** The effects of cAMP modulators on survival and maturation of vitrified-warmed GV oocytes with and without cumulus cell

	Total	MII	MI	GV	deg.	Survived oocytes (%)	Maturation (%)
COCs							
control	195	113	59	1	22	88.7 ^a^	57.9 ^a^
dbcAMP	205	139	46	1	19	90.7 ^a^	67.8 ^b^
IBMX	174	114	47	1	12	93.1 ^a^	65.5 ^a,b^
DOs							
control	232	84	114	8	26	88.8 ^a^	36.2 ^c^
dbcAMP	232	59	144	7	22	90.5 ^a^	25.4 ^d^
IBMX	245	79	133	7	26	89.4 ^a^	32.2 ^c,d^

Values with different superscripts are statistically different from each other (*p* < .05)

*cAMP* Cyclic adenosine monophosphate, *GV* Germinal vesicle, *COC* Cumulus-oocyte complex, *DO* Denuded oocyte, *dbcAMP* Dibutyryl-cAMP, *IBMX* 3-isobutyl-l-methylxanthine

### Chromatin integrity after the warming of GV oocytes

**Fig. 1:**
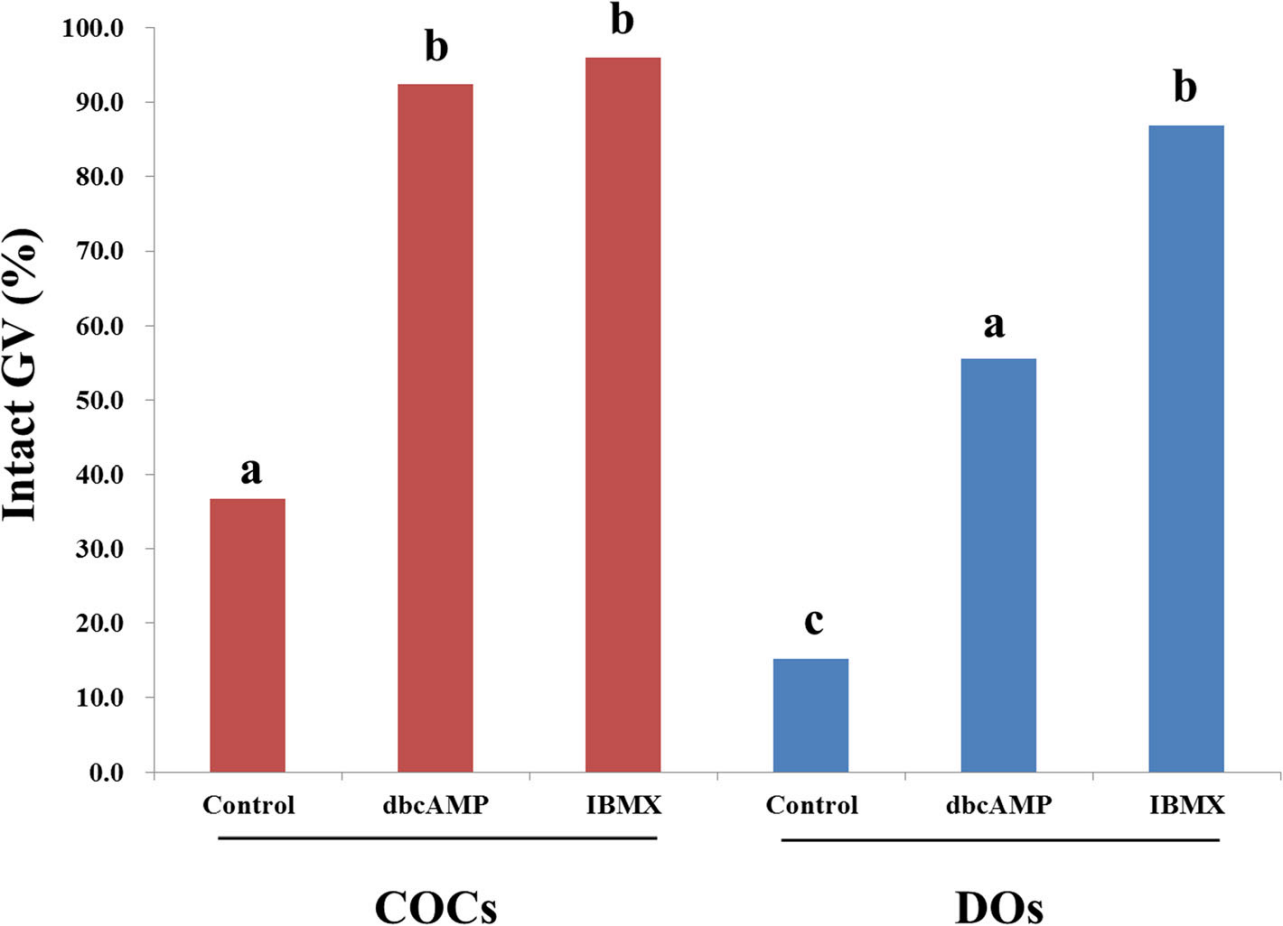
Proportions of germinal vesicle oocytes with intact chromatin integrity 1 h after warming. Values with different letters above the bar graph are statistically different from each other (*p* < 0.05). *GV* germinal vesicle, *COC* cumulus-oocyte complex, *DO* denuded oocyte, *dbcAMP* dibutyryl-cAMP, *IBMX* 3-isobutyl-l-methylxanthine

To determine the arrest status of GV oocytes immediately after warming, the chromatin integrity of oocytes was assessed 1 h after warming. GV oocytes were divided into intact GV oocytes and oocytes of pre-MI to MI stages. At least 25 GVs were compared for each group. When the control groups without the addition of cAMP modulators were compared, the proportion of oocytes arrested in the GV stage of the COC groups was significantly higher than that of the DO groups (Fig. 1). Within each of the COC and the DO groups, the percentage of oocytes in the intact GV stage was significantly higher in the groups treated with cAMP modulators. As a result, the effects of cAMP modulators on the inhibition of GV oocytes maturation in the early stages after warming were observed in both the COC groups and the DO groups. The addition of dbcAMP resulted in better cell cycle arrest in the COC groups than in the DO groups.

### Chromosome and spindle integrity of the MII oocytes

The chromosome and spindle integrity of the developed MII oocytes after 18 h of IVM was evaluated and divided into normal and abnormal findings. The representative results are presented in Additional file 1: Figure S1. There was no statistically significant difference in the proportion of oocytes showing normal chromosome and spindle organization among the six groups (Table 2). In all groups, over 90% of the oocytes expressed normal chromosome and spindle integrity.

**Table 2:** The effects of cAMP modulators on the chromosome and spindle organization on in vitro matured MII oocytes from vitrified-warmed GV oocytes with and without cumulus cell

	COCs			Dos		
	Control	dbcAMP	IBMX	Control	dbcAMP	IBMX
No. of oocytes examined	76	86	106	62	55	68
Normal (%)	71 (93.4%)	79 (91.9%)	99 (93.4%)	60 (96.8%)	52 (94.5%)	62 (91.2%)
Abnormal (%)	5 (6.6%)	7 (8.1%)	7 (6.4%)	2 (3.2%)	3 (5.5%)	6 (8.8%)

*cAMP* Cyclic adenosine monophosphate, *GV* Germinal vesicle, *COC* Cumulus-oocyte complex, *DO* Denuded oocyte, *dbcAMP* Dibutyryl-cAMP, *IBMX* 3-isobutyl-l-methylxanthine

### F-actin and expression

**Fig. 2:**
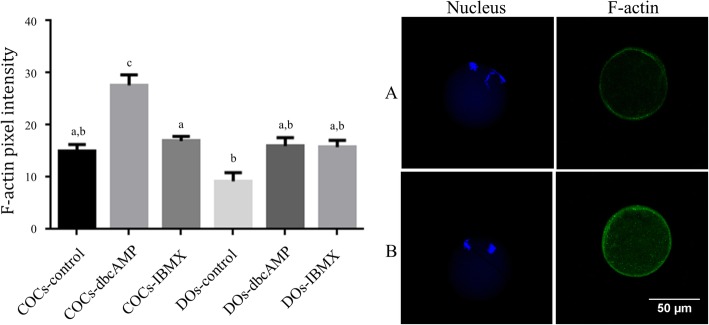
Effects of cAMP modulators on F-actin fluorescent intensity on in vitro matured MII oocytes from vitrified-warmed mouse GV oocytes with and without cumulus cell. The results are presented as means ± standard error of the mean. Values with different letters above the bar graph are statistically different from each other (*p* < 0.05). **a** Representative result of fluorescein intensity in the control group of cumulus-oocyte complex, **b** Representative result of fluorescein intensity in the dbcAMP treated group of cumulus-oocyte complex. *cAMP* cyclic adenosine monophosphate, *GV* germinal vesicle, *COC* cumulus-oocyte complex, *DO* denuded oocyte, *dbcAMP* dibutyryl-cAMP, *IBMX* 3-isobutyl-l-methylxanthine

We examined the fluorescence intensities of the F-actin in the cytoplasm and plasma membrane of the in vitro matured MII oocytes to investigate the mechanism of results shown in this study. ANOVA showed statistically significant differences between the 6 groups (Total degree of freedom = 123, F = 8.307, *p* < 0.001). The results of the quantitative analysis of the signal intensity are shown in Fig. 2. Among the COC groups, significantly more F-actin was observed in the cytoplasm of dbcAMP-treated groups than that of the other groups.

## Discussion

Our results suggest that treatment with dbcAMP before vitrification of the COCs of GV oocytes significantly improves the percentage of maturation after IVM. Treatment with cAMP modulators increases the intracellular cAMP level before vitrification and maintains cell cycle arrest immediately after warming. Although the effect of cAMP modulators on cell cycle arrest was observed in both the COC and the DO groups, the difference in the percentage of maturation indicates that the presence of cumulus cells plays an important role in the IVM process. Once the warmed GV oocytes were to mature, there was no difference in the chromosome and spindle integrity of the developed MII oocytes. The increased synthesis of F-actin which is a crucial component of cytoskeleton involved in spindle migration and chromosomal translocation was observed in the MII oocytes of dbcAMP-treated COC group.

Previous studies proved that CNP and its cognate receptor maintain oocyte meiotic arrest in mice [27]. This complex in cumulus cell increases cyclic guanosine monophosphate (cGMP) production and cGMP diffuse to oocytes through gap junctions and inhibits phosphodiesterase 3A. This leads to the elevation of cAMP levels in the oocyte [28]. The elevated cAMP inhibits the activity of protein kinase A, which suppresses the cyclin-dependent kinase 1/cyclin B complex activity to maintain meiotic arrest [29].

Accordingly, high cAMP concentration in the oocyte is essential for maintaining meiotic arrest, and when the concentration of cAMP decreases, meiosis resumes, and maturation occurs [30]. Maintaining adequate cAMP levels during oocyte maturation is an important requirement for chromatin transition and synchronization in the maturation processes of the nuclear and cytoplasmic components [31, 32].

GV oocytes are supplied with cGMP and cAMP through gap-junctions from multiple surrounding cumulus cells, and GV oocytes isolated from ovaries begin to resume meiosis as the supply of this cAMP decreases [28, 33]. According to the results of experiments conducted by Ezoe et al. using bovine oocytes, the intracellular cAMP levels immediately after warming were significantly reduced by the vitrification of COC [11]. Accordingly, raising intracellular cAMP using cAMP modulators immediately after oocyte separation from the ovary could play an important role in preventing the resumption of meiosis triggered by a decrease in intra-oocyte cAMP levels. Because oocytes are supplied with cAMP through the surrounding cumulus cells, the effect of the cAMP modulators on the net intracellular cAMP level would be greater in COC groups than in DO groups, and it may be beneficial to maintain the cAMP level homeostasis in the oocyte. In the present study, the cell cycle arrest effects of dbcAMP were higher in the COC groups than in DO groups, and the maturation rate of vitrified-warmed GV oocytes was significantly higher in COC groups than in DO groups. These results support the importance of COC in cell cycle arrest regulation and the maturation of immature oocytes.

Suppression of the oocyte cell cycle to GV state seems to be crucial to prevent cryodamage of DNA structure. The chromatin of the GV oocyte in the diplotene stage of prophase I is known to be less sensitive to cryoinjury because it is diffuse and surrounded by a nuclear membrane. However, when GV oocyte meiosis is reinitiated after removal from the inhibitory follicular environment, a cascade of nuclear maturation pathway is activated: germinal vesicle breakdown, chromatin condensation, meiotic spindle formation, and chromosome separation. In this process, tubulin polymerization begins when the first breaks appear in the envelope of the nucleus. These microtubules are highly sensitive to physical damage (e.g., cooling, exposure to cryoprotectants), leading to tubulin depolymerization and microtubular disassembly [34]. Therefore, maintaining the meiotic arrest of GV oocytes may help to minimize cryodamage caused by vitrification, and it is postulated that this protective effect may improve the maturation.

Although there was no difference in the chromosome and spindle integrity of the developed MII oocytes, damage of chromosomes and microstructures in the oocyte resulting from vitrification may lead to a decrease in the synthesis of components necessary for cell division. In this respect, we observed the expression of F-actin in mature oocytes. F-actin is involved in spindle migration and chromosomal translocation. Chromosomal translocation during oocyte meiosis is a coordinated process of F-actin and microtubule [35–37]. Therefore, it is noteworthy that the increase in expression of F-actin in the COC group treated with dbcAMP reflects better potency of spindle migration and chromosomal translocation in these oocytes.

In general, the goal of immature oocyte cryopreservation is to preserve the structural and functional integrity of COCs as a whole, including gap junctions. Meiosis resumption induces blocking of gap junction via phosphorylation of gap junction proteins [38]. This premature breakdown of COC gap junctions leads to loss of cumulus cell metabolites which contribute to the mature cytoplasm of the oocyte [7]. Therefore, it would be favorable if intact COCs could be cryopreserved without the initiation of meiosis resumption. As reflected in these theories, recent studies have shown that increasing cAMP levels of COCs using cAMP modulators within 1–2 h after oocyte retrieval increases COC gap junction communication and prevents precocious oocyte maturation, which improves developmental competence of the oocyte [7, 39]. Unfortunately, the connections between the oocyte and cumulus cells are easily damaged after the freezing and warming of intact COCs. Furthermore, the penetration of cryoprotective agents could be much more effective and faster in denuded oocytes compared to intact COCs, which subsequently affects the morphological, functional integrity, and survival of the cryopreserved oocytes. In the present study, the COC groups showed a higher percentage of maturation than the DO groups. The difference in maturation rate between the COC and the DO groups was more pronounced with the effect of the cAMP modulator. Reflecting these results, cryopreservation of GV oocytes in the COC state is more effective than cryopreservation in DO state, and this effect would be enhanced when cAMP modulator is treated.

The limitation of this study is that we could not compare the difference in the fertilization rate and development of the embryo from the acquired mature oocyte. The acquired mature oocytes in our experiment had poor fertilization capacity and the differences could not be accessed. Further study to improve the fertilization potential by modifying the cAMP modulator supplement protocols is planned to overcome this limitation. There have been studies on preserving the developmental potency of frozen oocytes by maintaining cell cycle arrest using a cAMP modulator, but few studies have analyzed the difference of microstructures involved in the cell division process. In this aspect, we analyzed the expression of substances involved in cell division such as F-actin and observed significant differences. In the future, further analysis to reveal the more fundamental mechanisms causing these differences may be needed.

In conclusion, increasing the intracellular cAMP level by administering cAMP modulators before vitrification maintains the cell cycle arrest by maintaining the level of cAMP in the oocyte immediately after warming. This process may facilitate oocyte maturation after IVM by preventing cryodamage of the oocyte and synchronizing maturation between nuclear and cytoplasmic components. The role of cumulus cells appears to be essential for this mechanism. Further studies are needed to improve the fertilization rate and developmental competence of embryos.

## Supplementary information

**Additional file 1: Figure S1.** Analysis of the chromosome and spindle organization of the developed MII oocyte in the six experimental groups. In abnormal findings, chromosome and spindle alignment are dislocated compared to normal findings. To distinguish between the two cases, the color settings of the α-tubulin are adjusted differently.

## Data Availability

The datasets used and/or analyzed during the current study are available from the corresponding author on reasonable request.

## References

[1] Cobo A, Diaz C (2011). Clinical application of oocyte vitrification: a systematic review and meta-analysis of randomized controlled trials. Fertil Steril.

[2] Pfeifer S, Gldberg J, McClure R, Lobo R, Thomas M, Widra E (2013). Practice committees of American Society for Reproductive Medicine; society for assisted reproductive technology. Mature oocyte cryopreservation: a guideline. Fertil Steril.

[3] Chian RC, Lim JH, Tan SL (2004). State of the art in in-vitro oocyte maturation. Curr Opin Obstet Gynecol.

[4] Lee JA, Barritt J, Moschini RM, Slifkin RE, Copperman AB (2013). Optimizing human oocyte cryopreservation for fertility preservation patients: should we mature then freeze or freeze then mature?. Fertil Steril.

[5] Sánchez F, Lolicato F, Romero S, De Vos M, Van Ranst H, Verheyen G (2017). An improved IVM method for cumulus-oocyte complexes from small follicles in polycystic ovary syndrome patients enhances oocyte competence and embryo yield. Hum Reprod.

[6] Yang L, Wei Q, Li W, Ge J, Zhao X, Ma B (2016). C-type natriuretic peptide improved vitrified-warmed mouse cumulus oocyte complexes developmental competence. Cryobiology.

[7] Albuz F, Sasseville M, Lane M, Armstrong D, Thompson J, Gilchrist R (2010). Simulated physiological oocyte maturation (SPOM): a novel in vitro maturation system that substantially improves embryo yield and pregnancy outcomes. Hum Reprod.

[8] Nogueira D, Ron-El R, Friedler S, Schachter M, Raziel A, Cortvrindt R (2006). Meiotic arrest in vitro by phosphodiesterase 3-inhibitor enhances maturation capacity of human oocytes and allows subsequent embryonic development. Biol Reprod.

[9] Shu YM, Zeng HT, Ren Z, Zhuang GL, Liang XY, Shen HW (2008). Effects of cilostamide and forskolin on the meiotic resumption and embryonic development of immature human oocytes. Hum Reprod.

[10] Vanhoutte L, Nogueira D, Dumortier F, De Sutter P (2009). Assessment of a new in vitro maturation system for mouse and human cumulus-enclosed oocytes: three-dimensional prematuration culture in the presence of a phosphodiesterase 3-inhibitor. Hum Reprod.

[11] Ezoe K, Yabuuchi A, Tani T, Mori C, Miki T, Takayama Y (2015). Developmental competence of vitrified-warmed bovine oocytes at the germinal-vesicle stage is improved by cyclic adenosine monophosphate modulators during in vitro maturation. PLoS One.

[12] Coticchio G, Bromfield JJ, Sciajno R, Gambardella A, Scaravelli G, Borini A (2009). Vitrification may increase the rate of chromosome misalignment in the metaphase II spindle of human mature oocytes. Reprod BioMed Online.

[13] Azoury J, Lee KW, Georget V, Rassinier P, Leader B, Verlhac MH (2008). Spindle positioning in mouse oocytes relies on a dynamic meshwork of actin filaments. Curr Biol.

[14] Nogueira D, Albano C, Adriaenssens T, Cortvrindt R, Bourgain C, Devroey P (2003). Human oocytes reversibly arrested in prophase I by phosphodiesterase type 3 inhibitor in vitro. Biol Reprod.

[15] Nogueira D, Cortvrindt R, De Matos D, Vanhoutte L, Smitz J (2003). Effect of phosphodiesterase type 3 inhibitor on developmental competence of immature mouse oocytes in vitro. Biol Reprod.

[16] Luciano A, Pocar P, Milanesi E, Modina S, Rieger D, Lauria A (1999). Effect of different levels of intracellular cAMP on the in vitro maturation of cattle oocytes and their subsequent development following in vitro fertilization. Mol Reprod Dev.

[17] Funahashi H, Cantley TC, Day BN (1997). Synchronization of meiosis in porcine oocytes by exposure to dibutyryl cyclic adenosine monophosphate improves developmental competence following in vitro fertilization. Biol Reprod.

[18] Fasano G, Demeestere I, Englert Y (2012). In-vitro maturation of human oocytes: before or after vitrification?. J Assist Reprod Genet.

[19] Song WY, Peng ZF, Chen XM, Jin HX, Yao GD, Shi SL (2016). Effects of Vitrification on outcomes of in VivoMature, in vitro-mature and immature human oocytes. Cell Physiol Biochem.

[20] Kasapi E, Asimakopoulos B, Chatzimeletiou K, Petousis S, Panagiotidis Y, Prapas N (2017). Vitrification of human germinal vesicle oocytes: before or after in vitro maturation?. Int J Fertil Steril.

[21] Chung HM, Hong SW, Lim JM, Lee SH, Cha WT, Ko JJ (2000). In vitro blastocyst formation of human oocytes obtained from unstimulated and stimulated cycles after vitrification at various maturational stages. Fertil Steril.

[22] Wu J, Zhang L, Wang X (2001). In vitro maturation, fertilization and embryo development after ultrarapid freezing of immature human oocytes. Reproduction.

[23] Cho WK, Stern S, Biggers JD (1974). Inhibitory effect of dibutyryl cAMP on mouse oocyte maturation in vitro. J Exp Zool.

[24] Richter JD, McGaughey RW (1981). Patterns of polypeptide synthesis in mouse oocytes during germinal vesicle breakdown and during maintenance of the germinal vesicle stage by dibutyryl cAMP. Dev Biol.

[25] Bornslaeger EA, Mattei P, Schultz RM (1986). Involvement of cAMP-dependent protein kinase and protein phosphorylation in regulation of mouse oocyte maturation. Dev Biol.

[26] Beaujean N, Taylor JE, McGarry M, Gardner JO, Wilmut I, Loi P (2004). The effect of interspecific oocytes on demethylation of sperm DNA. Proc Natl Acad Sci.

[27] Zhang M, Su YQ, Sugiura K, Xia G, Eppig JJ (2010). Granulosa cell ligand NPPC and its receptor NPR2 maintain meiotic arrest in mouse oocytes. Science.

[28] Zhang M, Ouyang H, Xia G (2009). The signal pathway of gonadotrophins-induced mammalian oocyte meiotic resumption. Mol Hum Reprod.

[29] Mehlmann LM (2005). Stops and starts in mammalian oocytes: recent advances in understanding the regulation of meiotic arrest and oocyte maturation. Reproduction.

[30] Sela-Abramovich S, Edry I, Galiani D, Nevo N, Dekel N (2006). Disruption of gap junctional communication within the ovarian follicle induces oocyte maturation. Endocrinology.

[31] Smitz JE, Thompson JG, Gilchrist RB (2011). The promise of in vitro maturation in assisted reproduction and fertility preservation. Semin Reprod Med.

[32] Luciano AM, Franciosi F, Modina SC, Lodde V (2011). Gap junction-mediated communications regulate chromatin remodeling during bovine oocyte growth and differentiation through cAMP-dependent mechanism (s). Biol Reprod.

[33] Gilchrist RB (2010). Recent insights into oocyte–follicle cell interactions provide opportunities for the development of new approaches to in vitro maturation. Reprod Fertil Dev.

[34] Mandelbaum J, Anastasiou O, Levy R, Guérin J, De Larouziere V, Antoine J (2004). Effects of cryopreservation on the meiotic spindle of human oocytes. Eur J Obstet Gynecol Reprod Biol.

[35] Longo E, Stamato F, Ferreira R, Tapia O (1985). The catalytic mechanism of serine proteases II: the effect of the protein environment in the α-chymotrypsin proton relay system. J Theor Biol.

[36] Maro B, Johnson MH, Webb M, Flach G (1986). Mechanism of polar body formation in the mouse oocyte: an interaction between the chromosomes, the cytoskeleton and the plasma membrane. J Embryol Exp Morpholog.

[37] Burdyniuk M, Callegari A, Mori M, Nédélec F, Lénárt P (2018). F-actin nucleated on chromosomes coordinates their capture by microtubules in oocyte meiosis. J Cell Biol.

[38] Conti M, Hsieh M, Park JY, Su YQ (2006). Role of the epidermal growth factor network in ovarian follicles. Mol Endocrinol.

[39] Zeng HT, Ren Z, Guzman L, Wang X, Sutton-McDowall ML, Ritter LJ (2013). Heparin and cAMP modulators interact during pre-in vitro maturation to affect mouse and human oocyte meiosis and developmental competence. Hum Reprod.

