# Supplementary figures and images for: Effects of cyclic adenosine monophosphate modulators on maturation and quality of vitrified-warmed germinal vesicle stage mouse oocytes
Source: Reprod Biol Endocrinol. 2020 Jan 20;18:5. doi: 10.1186/s12958-020-0566-8 (PMC6971999; doi:10.1186/s12958-020-0566-8)

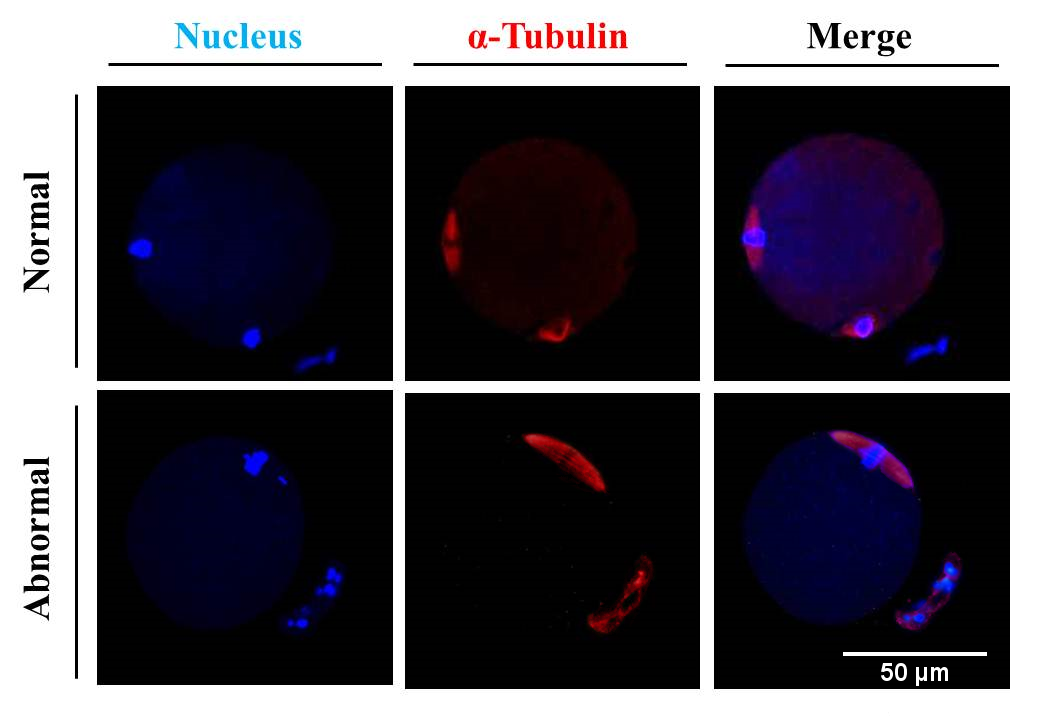

Supplement: Supplementary file 1 — Additional file 1: Figure S1. Analysis of the chromosome and spindle organization of the developed MII oocyte in the six experimental groups. In abnormal findings, chromosome and spindle alignment are dislocated compared to normal findings. To distinguish between the two cases, the color settings of the α-tubulin are adjusted differently. [file 12958_2020_566_MOESM1_ESM.tif]
